# Lumbar artery aneurysm complicated by a fistula between the aneurysm and the duodenum in a patient with Leriche syndrome: A case report

**DOI:** 10.1016/j.ijscr.2020.11.087

**Published:** 2020-11-19

**Authors:** Toru Imagami, Masaki Sakamoto, Hisanori Kani, Masao Tadakoshi

**Affiliations:** a Department of Surgery, Nagoya Tokushukai General Hospital, Kasugai City, Japan; b Department of Cardiovascular Surgery, Nagoya Tokushukai General Hospital, Kasugai City, Japan

**Keywords:** CT, computed tomography, TAE, transcatheter arterial embolization, Lumbar artery aneurysm, Leriche syndrome, Arterio-enteric fistula

## Abstract

**Introduction:**

Various collateral pathways maintain blood flow to the lower extremities in patients with Leriche syndrome. The occurrence of true aneurysms in the lumbar artery—a component of an extensive collateral circulation network in patients with Leriche syndrome—is extremely rare.

**Presentation of case:**

A 73-year-old man with Leriche syndrome was diagnosed with lumbar artery aneurysm complicated by a duodenal fistula. The patient underwent endovascular repair, surgical duodenal fistula closure, and debridement of the aneurysm wall until coil exposure.

**Discussion:**

With the same mechanism, patients with aortic occlusive disease may develop an aneurysm and arterio-enteric fistula in the collateral circulation. Combination of treatments may be important for hemostasis, control of infection, and maintaining adequate distal perfusion.

**Conclusion:**

Endovascular embolization can control bleeding as well as serve as a landmark for the debridement of contaminated aneurysm. Surgical fistula closure and aneurysm-wall debridement are useful for control of local infection.

## Highlights

• The arterio-enteric fistula between lumbar artery and duodenum is rare pathology.
• Aneurysm may occur in the collateral circulation in patients with Leriche syndrome.
• Endovascular embolization can control hemorrhage immediately.
• Fistula closure and debridement is recommended for control of local infection.
• Endovascular embolization can serve as a landmark for the debridement of aneurysm.

## 1 Introduction

Various collateral pathways maintain blood flow to the lower extremities in patients with Leriche syndrome [1]. The occurrence of true aneurysms in the lumbar artery—a component of an extensive collateral circulation network in patients with Leriche syndrome—is extremely rare. To the best our knowledge, this is the first report of an arterio-enteric fistula occurring between a lumbar-artery aneurysm and the duodenum.

Here, we present a rare case of lumbar-artery aneurysm complicated by a duodenal fistula in a patient with Leriche syndrome. Endovascular repair was performed combined with open surgery. A good outcome was achieved in this challenging case; therefore, the report of this clinical experience could contribute to the development of treatment options for arterio-enteric fistula. This work has been reported in line with the SCARE criteria [2].

## 2 Presentation of case

A 73-year-old man presenting with vomiting blood was referred to a local hospital. He had a medical history of hypertension, diabetes mellitus, ischemic heart disease, chronic obstructive pulmonary disorder, and Leriche syndrome and a surgical history of coronary artery bypass graft and lung resection for lung cancer. He had been prescribed antihypertensives, oral diabetes medicine and antiplatelet medicine with aspirin. He was a heavy smoker with a Brinkman index of 1400. His claudication distance was about 50 m. There was no difference between left and right.

**Fig. 1:**
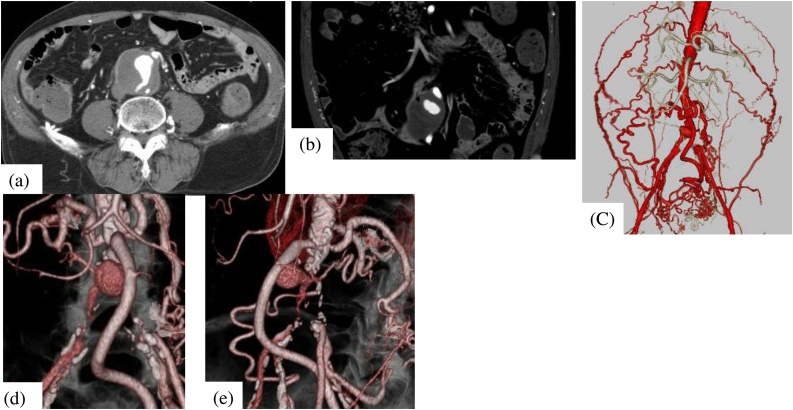
The CT findings at outpatient. (a) On contrast enhanced CT, a true aneurysm was identified near the right side of the terminal aorta. (b) The aneurysm and duodenum were in contact. Air sign was identified within the wall of the aneurysm. (c) Contrast enhanced CT revealed collateral vessels that had developed throughout the body. (d)(e) Contrast enhanced CT revealed the occlusion of the aortic bifurcation. An aneurysm was found near the terminal aorta. The feeding artery of the aneurysm was suspected as right common iliac artery.

Laboratory investigations revealed a hemoglobin level of 11.8 g/dL, white blood cell count of 11,000/mm3, platelet count of 101,000/mm3, urea nitrogen level of 23.8 mg/dL, creatinine 0.94 mg/dL, and C-reactive protein level of 3.06 mg/dL. Liver function and coagulation profiles were within the normal range. Emergency gastroscopy identified blood retention in the second-to-third portion of the duodenum, but the source of bleeding could not be identified. The patient was transferred to our hospital for further treatment. Contrast-enhanced computed tomography (CT) revealed the occlusion of the terminal aorta and collateral vessels that had developed throughout the body (Fig. 1). A true aneurysm was identified near the right side of the terminal aorta. The aneurysm and duodenum were in contact, and the fat plane between them disappeared. Air was identified within the wall of the aneurysm. On the basis of these findings, we diagnosed the patient with aneurysm duodenal fistula.

Consultation between surgeons and cardiovascular surgeons concluded that transcatheter arterial embolization (TAE) and open surgical fistula resection with debridement of the aneurysm was required to control hemorrhage and infection. After written informed consent was obtained, cardiovascular surgeons first performed endovascular repair to control hemorrhaging, and then surgeons performed open surgery.

**Fig. 2:**
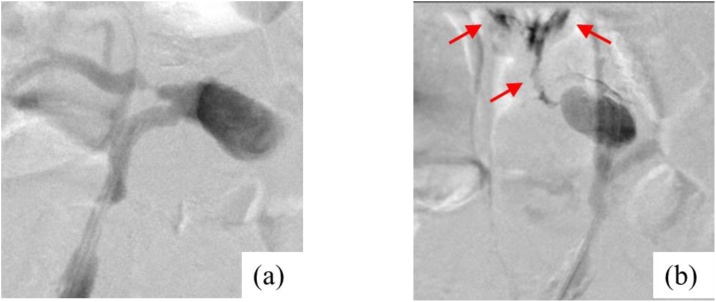
The findings of angiography. (a) Angiography from right common iliac artery showed an aneurysm and its inflow and outflow vessels. The etiological artery was considered lumbar artery that developed as collateral pathway from the aorta to the right common iliac artery. (b) Angiography revealed fistula and duodenum (arrow).

The right femoral artery was cannulated using a 5-Fr long-sheath catheter with the patient under local anesthesia. Angiography from the right common iliac artery revealed blood flow to be reversed and directed toward the aneurysm (Fig. 2). Angiography from the inflow vessel revealed that contrast agent flowed from the aneurysm into the lumbar artery and, simultaneously, into the duodenum, confirming aneurysm of the lumbar artery leading to duodenal fistula. Although it was challenging to cannulate the aneurysm, TAE with a coil and an 8-mm Amplatzer Vascular Plug II was successfully performed and final angiography confirmed complete isolation of the aneurysm. The total operative time was 110 min and the hemodynamic state was stable intraoperatively.

**Fig. 3:**
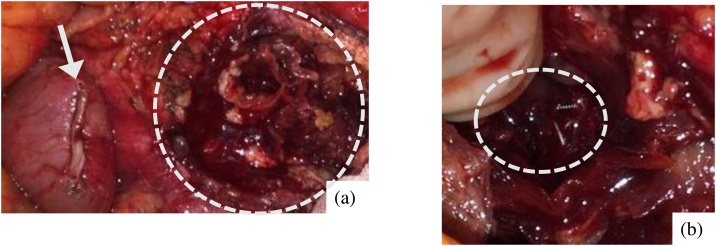
The intraoperative findings. (a) The duodenal fistula was closed using linear stapler (arrow). Debridement of the wall of the aneurysm wall was performed (circle). (b) The coil used for embolization was exposed (circle).

The following day, the aneurysm was accessed via median incision under general anesthesia. Dissection around the aneurysm was easily achieved, enabling the fistula between the aneurysm and the duodenum to be identified and closed using a linear stapler (Fig. 3a). Debridement of the wall of the aneurysm was performed until the coil was exposed (Fig. 3b), and then lavage was performed with a large amount of saline. No active bleeding occurred at the aneurysm debridement due to TAE. Operative time was 71 min.

The postoperative course was uneventful. Follow-up CT 3 days postoperatively confirmed the absence of infection; thus, antibiotic administration was terminated. Circulation to the lower extremities was maintained via other collateral pathways. The patient discharged 12 days postoperatively with no physical symptoms. Follow-up CT 1 month postoperatively revealed no abnormal findings, and follow-up is ongoing through the outpatient department presently, 1 year postoperatively. His claudication was unchanged after the intervention.

## 3 Discussion

Leriche was the first to correlate the clinical triad of claudication, erectile dysfunction, and decreased distal pulses with thrombotic obliteration of the aortic bifurcation and coined this entity as “Leriche syndrome” [3,4]. Lumbar arteries originate as small branches of the abdominal aorta, iliac lumbar artery, or median artery, and are approximately 2.9 mm in diameter [5]. In the present case, the collateral pathways formed a complex network; we identified an aneurysm of the developed collateral pathways between the lumbar artery and iliac artery.

While aortoiliac occlusive disease and aneurysms share a common baseline pathophysiology involving atherosclerotic changes, co-existence of the two conditions is rare [6,7]. Aneurysms that are caused by hemodynamics occur in response to changes in the blood flow, whereas true pancreaticoduodenal artery aneurysms are considered to arise from an increased flow through the pancreatic arcade [8]. As with pancreaticoduodenal artery aneurysm, we hypothesized that—in the present case—increased blood flow to the small and fragile collateral vessels caused a true lumbar artery aneurysm. Aneurysms in patients with aortic occlusive disease may also occur through this mechanism; therefore, this case presents an important example of aneurysms in the collateral pathways of aortic occlusive disease.

Arterio-enteric fistulas are defined as abnormal connections between an artery and the gastrointestinal tract [9], and it is mostly observed between the aorta and the duodenum. For the diagnosis, CT findings included the disappearance of the fat plane between the aneurysm and the duodenum, air in the retroperitoneum or aortic wall, and contrast enhancement within the duodenum [10]. All three were observed in the present case. In the present case, contrast-enhanced CT did not show extravasation; however, selective angiography from the feeding vessel to the aneurysm revealed extravasation into the duodenum. Angiography can provide valuable information on the complex anatomy of the collateral circulation pathways and should thus be considered when arterio-enteric fistula is suspected, especially in cases with complicated collateral pathways.

Traditionally, management of arterio-enteric fistula has focused on controlling hemorrhage, infection, and maintaining adequate distal perfusion [11,12]. The time interval between herald bleeding and massive hemorrhage ranges from hours to months [13], so immediate hemostasis is required. Endovascular repair offers a rapid control of hemorrhage in unstable patients [12]; therefore, we employed endovascular repair.

Disadvantage of endovascular repair is that bowel defects cannot be repaired [14], and, thus, bacterial growth and persistent infections ensue. The implantation of new prosthetic material into an already or potentially infected field without eradicating the source of infection has been questioned for the long-term safety and efficacy [12]. Therefore, clinicians should consider the degree of contamination when planning therapeutic approaches for arterio-intestinal fistula [15]. However, accurate determination of the degree of contamination is difficult preoperatively, meaning that surgical procedures should include measures to control infection, such as omental coverage or extensive debridement. In the present case, we performed the debridement of the contaminated hematoma until the coil was exposed. Performing TAE prior to open surgery enabled to extensive debridement of the contaminated aneurysm while avoiding bleeding from the aneurysm. It has previously been reported that a 1-week postoperative antibiotic regimen should be administered for arterio-enteric fistula, even if cultures appear negative [16]; however, we withdrew antibiotic administration 3 days postoperatively because we considered the extensive debridement could prevent local infection. Further research is required to determine the appropriate duration of antibiotic administration, especially in patients undergoing infection-control surgery.

Revascularization needs to be considered when endovascular embolization is performed for collateral arteries. Femorofemoral bypass was planned if lower-limb ischemia developed; however, the patient did not complain of lower-limb ischemia postoperatively. Doppler sonographic flow measurements of the contribution of collateral pathways may provide beneficial diagnostic information [1].

## 4 Conclusion

Endovascular embolization combined with surgical fistula closure and aneurysm-wall debridement for lumbar artery aneurysm complicated with duodenal fistula resulted in a good outcome for a patient with Leriche syndrome. This case provides the following important findings: 1) patients with aortic occlusive diseases may suffer from true aneurysm in developed collateral pathways such as the lumbar artery; 2) endovascular embolization can control bleeding as well as serve as a landmark for the debridement of contaminated aneurysm with intestinal fistula; 3) surgical fistula closure and aneurysm-wall debridement appear sufficient to control local infection.

## Declaration of Competing Interest

The authors have no conflict of interest to declare.

## Sources of funding

This study did not receive any specific grant from funding agencies in the public, commercial, or not-for-profit sectors.

## Ethical approval

This study was approved by the ethics committee in Nagoya Tokushukai General Hospital.

## Consent

Written informed consent was obtained from patient for publication of this case report and accompanying images.

## Author contribution

TI, ST and MS performed open surgery. MT performed endovascular repair. TI drafted the manuscript. HK participated in the correction of the manuscript. All authors approved the final manuscript.

## Registration of research studies

No research study involved in this case report. Not applicable.

## Guarantor

Toru Imagami.

## Provenance and peer review

Not commissioned, externally peer-reviewed.
